# Calculating Sensitivity, Specificity, and Predictive Values for Correlated Eye Data

**DOI:** 10.1167/iovs.61.11.29

**Published:** 2020-09-16

**Authors:** Gui-Shuang Ying, Maureen G. Maguire, Robert J. Glynn, Bernard Rosner

**Affiliations:** 1 Center for Preventive Ophthalmology and Biostatistics, Department of Ophthalmology, Perelman School of Medicine, University of Pennsylvania, Philadelphia, Pennsylvania, United States; 2 Division of Preventive Medicine and the Channing Lab, Department of Medicine, Brigham and Women's Hospital, Boston, Massachusetts, United States

**Keywords:** sensitivity, specificity, predictive values, correlated eye data

## Abstract

**Purpose:**

To describe and demonstrate appropriate statistical approaches for estimating sensitivity, specificity, predictive values and their 95% confidence intervals (95% CI) for correlated eye data.

**Methods:**

We described generalized estimating equations (GEE) and cluster bootstrap to account for inter-eye correlation and applied them for analyzing the data from a clinical study of telemedicine for the detection of retinopathy of prematurity (ROP).

**Results:**

Among 100 infants (200 eyes) selected for analysis, 20 infants had referral-warranted ROP (RW-ROP) in both eyes and 9 infants with RW-ROP only in one eye based on clinical eye examination. In the per-eye analysis that included both eyes of an infant, the image evaluation for RW-ROP had sensitivity of 83.7% and specificity of 86.8%. The 95% CI's from the naïve approach that ignored the inter-eye correlation were narrower than those of the GEE approach and cluster bootstrap for both sensitivity (width of 95% CI: 22.4% vs. 23.2% vs. 23.9%) and specificity (11.4% vs. 12.5% vs. 11.6%). The 95% CIs for sensitivity and specificity calculated from left eyes and right eyes separately were wider (35.2% and 30.8% respectively for sensitivity, 25.4% and 17.3% respectively for specificity).

**Conclusions:**

When an ocular test is performed in both eyes of some or all of the study subjects, the statistical analyses are best performed at the eye-level and account for the inter-eye correlation by using either the GEE or cluster bootstrap. Ignoring the inter-eye correlation results in 95% CIs that are inappropriately narrow and analyzing data from two eyes separately are not efficient.

Diagnostic and screening tests play an important role in the detection and management of eye diseases, as well as in laboratory research. Evaluation of advances in ocular imaging technologies, telemedicine, machine learning, artificial intelligence technologies, biomarkers, and statistical prediction models or risk scores (broadly referred to as “ocular tests” within this paper) often involves determining whether a specific condition is present. Examples of symptoms, signs, or laboratory values to identify or predict a specific condition include cup-to-disc ratio to identify glaucoma, large drusen to predict development of late age-related macular degeneration, or the level of the antibody SSa (Ro) to diagnose Sjogren Syndrome. Before a new ocular test can be adopted for clinical use, its accuracy in identifying the specific condition must be evaluated in a sample from the targeted population, using performance indices, including sensitivity, specificity, and predictive values.^1^ Because ocular measures are commonly taken from both eyes of a subject, thereby generating correlated eye data, statistical analyses for evaluating the accuracy of the ocular test need to account for the correlation. In this paper, we describe and demonstrate appropriate statistical approaches for estimating these performance indices and their 95% confidence intervals (CIs). In addition, we consider whether the presence of the condition should be evaluated per subject or per eye.

We start with a general introduction of performance indices (sensitivity, specificity, and predictive values) and the calculations for their point estimates and 95% CIs when the data are independent (e.g. one test measure from each subject). We then describe statistical methods to account for inter-eye correlation when an ocular test is performed in both eyes of a subject. We demonstrate these statistical methods by analyzing the data from a clinical study of telemedicine for the detection of retinopathy of prematurity (ROP).

## Calculating Performance Indices for a Test Using Independent Data

### Sensitivity and Specificity

To evaluate the accuracy of a test, we use a sample of subjects who have results from both the test (T) and the reference standard procedure for determining disease status (D). We assume that the reference standard procedure is 100% accurate for determining disease presence (D+) or absence (D-). For a test that yields binary test results (i.e. test positive [T+] or negative [T-]), results from the test and the reference standard procedure can be summarized in a 2 × 2 Table (Table 1). For a test that provides continuous or ordinal values, a 2 × 2 table can be made by applying a cutpoint to dichotomize the test results into test positive or negative. The choice of a cutpoint depends on several factors, including the relative harms and costs of missing true disease and of falsely identifying disease as present when it is not.^2^

**Table 1.** A 2 × 2 Table for Comparing Results from a Test and a Reference Standard Procedure

	True Disease Status From Reference Standard Procedure	
Test Result	Absent (D−)	Present (D+)
Negative (T−)	n_00_	n_10_
Positive (T+)	n_01_	n_11_
Total	n_0_	n_1_

Two basic measures of test accuracy, sensitivity and specificity, can be estimated from the values in Table 1. Sensitivity is the test's ability to detect the disease when the disease is present (i.e. Sensitivity *(Se) =P(T+|D+) = n_11_ / n_1_*) or, in words, the probability of a positive test result given that the disease is present. Specificity is the test's ability to exclude the disease when the disease is absent (i.e. Specificity *(Sp) = P(T-|D-) = n_00_ / n_0_*) or, in words, the probability of a negative test result given that the disease is absent.

To determine the uncertainty of the estimates of sensitivity and specificity, 95% CIs are calculated. For independent large samples, the 95% CIs can be calculated using the normal approximation^3^:

$$\begin{array}{c} Se\pm 1.96\times \sqrt{\frac{Se\times (1-Se)}{n_{1}}}\\ Sp\pm 1.96\times \sqrt{\frac{Sp\times (1-Sp)}{n_{0}}} \end{array}$$

When the sample size is small or the sensitivity or specificity is close to 0 or 1 (e.g. *n*_1_ × *Se* × (1 − *Se*) or *n*_0_ × *Sp* × (1 − *Sp*) is less than 5), the normal approximation may not be accurate.^4^ Other methods,^4^ such as the Clopper-Pearson exact method or the Wilson method, should be used to provide better accuracy and to guarantee the 95% CIs are within the desired range of 0 to 1.^5^ The Clopper–Pearson interval provides an exact interval because it is directly based on the cumulative probabilities of the binomial distribution rather than an approximation to the binomial distribution. The Clopper–Pearson interval never has less than the nominal coverage (e.g. 95%), so it is usually conservative.^5^ The Wilson interval is an improvement over the normal approximation interval in that the actual coverage probability is closer to the nominal value. The Wilson method has good properties even for a small number of observations and/or an extreme alpha error level. Clopper-Pearson, Wilson, and other alternative intervals are available in most statistical packages, and further details on their implementation and performance are described elsewhere.^4^^,^^5^

### Positive and Negative Predictive Values

Sensitivity and specificity measure the intrinsic accuracy of a test and require that the status of disease is known. However, in clinical practice when using a test, the true disease status is usually unknown and we perform the test to inform us about the presence of the disease. When using a test, we need to know how well the test result predicts the presence or absence of disease. The positive predictive value (PPV) and negative predictive value (NPV) provide such information. The PPV is the probability that a positive test result correctly predicts the presence of disease, whereas NPV is the probability that a negative test result correctly predicts the absence of disease.

The PPV and NPV are dependent on both the underlying prevalence of disease in the population to be tested and the intrinsic accuracy (i.e. sensitivity and specificity) of the test. For any given test, when the disease prevalence is higher, the PPV is higher while the NPV is lower. For this reason, it is usually not appropriate to calculate the PPV and NPV directly from studies that oversample subjects with disease (such as a 1:1 case-control study that artificially sets the disease prevalence at 50%), because such studies do not reflect the true disease prevalence in the population that the test will be used in. Instead, using the following formula, the sensitivity (*Se*) and specificity (*Sp*) estimated from a case-control study can be applied to calculate the PPV and NPV of a test in a target population with disease prevalence (*P*), which is usually estimated from a separate study.

$$\begin{array}{c} PPV=\frac{Se\times P}{Se\times P+(1-Sp)\times (1-P)}\\ NPV=\frac{Sp\times (1-P)}{\left(1-Se)\times P+Sp\times (1-P\right)} \end{array}$$

To calculate 95% CIs for the PPV and NPV, their variances need to be determined using the following formula^6^:

$$\begin{array}{c} \begin{array}{c} Var\left(PPV\right)=\frac{{\left[P\times (1-Sp)\times (1-P)\right]}^{2}\times \frac{Se\times (1-Se)}{n_{1}}+{\left[P\times Se\times (1-P)\right]}^{2}\times \frac{Sp\times (1-Sp)}{n_{0}}}{{\left[Se\times P+(1-Sp)\times (1-P)\right]}^{4}}\\ Var\left(NPV\right)=\frac{\left[Sp\times \left(1-P\right)\times P\right){]}^{2}\times \frac{Se\times (1-Se)}{n_{1}}+{\left[(1-Se)\times (1-P)\times P\right]}^{2}\times \frac{Sp\times (1-Sp)}{n_{0}}}{{\left[(1-Se)\times P+Sp\times (1-P)\right]}^{4}} \end{array} \end{array}$$

where *P* is the prevalence of the disease of interest (assumed known), *Se* and *Sp* are the sensitivity and specificity of the test for detecting the disease of interest, *n_1_* and *n_0_* are the number of subjects with and without disease in the study for calculating the sensitivity and specificity, respectively.

With variances of PPV and NPV calculated using the above formula, 95% CIs for PPV and NPV can be calculated as:

$$\begin{array}{c} PPV\pm 1.96\sqrt{Var(PPV)}\\ NPV\pm 1.96\sqrt{Var(NPV)} \end{array}$$

When the PPV or NPV is close to 0 or 1, their 95% CIs calculated using the normal approximation can be out of the desired range of 0 to 1. The logit transformation,^6^ as described below, can be used to calculate the 95% CIs to guarantee that they fall between 0 and 1.

$$\begin{array}{c} logit\left(PPV\right)=\log\left(\frac{PPV}{1-PPV}\right)\\ logit\left(NPV\right)=\log\left(\frac{NPV}{1-NPV}\right) \end{array}$$

The variance of the logit(PPV) and logit(NPV) can be calculated as follows:

$$\begin{array}{c} Var\left(\mathrm{logit}\left(PPV\right)\right)=\left[\frac{1-Se}{Se}\right].\frac{1}{n_{1}}+\left[\frac{Sp}{1-Sp}\right].\frac{1}{n_{0}}\\ Var\left(\mathrm{logit}\left(NPV\right)\right)=\left[\frac{Se}{1-Se}\right].\frac{1}{n_{1}}+\left[\frac{1-Sp}{Sp}\right].\frac{1}{n_{0}} \end{array}$$

The 95% CI for PPV is calculated as follows:

$$\begin{array}{c} \begin{array}{c} \left[\frac{e^{\mathrm{logit}\left(PPV\right)-1.96\sqrt{Var(\mathrm{logit}(PPV))}}}{1+e^{\mathrm{logit}\left(PPV\right)-1.96\sqrt{Var(\mathrm{logit}(PPV))}}},\frac{e^{\mathrm{logit}\left(PPV\right)+1.96\sqrt{Var(\mathrm{logit}(PPV))}}}{1+e^{\mathrm{logit}\left(PPV\right)+1.96\sqrt{Var(\mathrm{logit}(PPV))}}}\right] \end{array} \end{array}$$

The 95% confidence interval for NPV can be calculated similarly.

An SAS macro for calculating the PPV, NPV, and their 95% CIs using both the normal approximation and the logit transformation for a given set of values for sensitivity, specificity, and prevalence of disease is provided in Appendix 1.

## Performance Indices of an Ocular Test for Correlated Eye Data

### Determination of Ocular Test Performance at Eye Level

As most eye diseases can be bilateral, ocular tests are often performed in both eyes of a subject, yielding correlated eye data. To maximize the use of the available data, sensitivity and specificity can be calculated at the eye-level (i.e. using the eye as the unit of analysis), whereas the correlation between the two eyes (i.e. the inter-eye correlation) is accounted for. When each subject contributes both eyes for the study, the standard method previously described above for a sample of independent observations provides unbiased point estimates of sensitivity and specificity for correlated eye data. However, calculating their 95% CIs needs to account for the inter-eye correlation. Ignoring the inter-eye correlation (i.e. treating data from two eyes of the same subject in the same way as data from two eyes from two different subjects) yields 95% CIs that are too narrow. When some subjects contribute only one eye whereas other subjects contribute both eyes for the study, using the previously described analysis approaches for independent samples that ignore the inter-eye correlation could lead to biased estimates for sensitivity and specificity and their 95% CIs.

One approach for adjusting for the inter-eye correlation is through use of generalized estimating equations (GEEs).^7^ In applying the GEE approach to estimating sensitivity and specificity, the ocular test result for each eye (T+ or T-) is modeled as the outcome variable, the variable for true eye disease status (D+ or D-) from the reference standard procedure is considered as a predictor, and the logit link is used. By convention, a positive test result is assigned a value of 1 and a negative value is assigned a value of 0, and likewise for disease presence. One way to use the GEE approach is to specify in the statistical software code that the data are “independent” and rely on the approach's robust estimator to provide accurate variance estimates to be used for calculation of 95% CIs. This specification is often the default option for procedures using GEE. Although this appears to be an incorrect choice for correlated data, this method works well for the case of modeling a 2 × 2 table. More detailed descriptions of the GEE method for accounting for inter-eye correlation in analyzing categorical ocular measures may be found elsewhere.^8^ The SAS code for the calculation of the 95% CI of sensitivity and specificity using GEE is given in Appendix 2. Of note, in fitting GEE using PROC GENMOD in SAS, the DESCENDING option was specified so that it models the probability of disease. In R, GEE modeling can be performed by using the function geeglm() of the “geepack” package or using the function gee() of the “GEE” package. When running these GEE functions in R, it is important to first sort the data by subject ID so that data from two eyes of the same subject are adjacent to each other; otherwise, the data from the two eyes of a subject will be analyzed as independent. In SAS, sorting the data by subject ID is not needed for GEE.

Another approach to account for the inter-eye correlation is the cluster bootstrap. Various bootstrap approaches have been proposed for clustered data.^9^ Bootstrapping is a resampling technique involving computing a statistic of interest (e.g. sensitivity, specificity, predictive values, etc.) repeatedly based on a large number of random samples drawn from the original sample, so that the variability of the statistic of interest can be determined. The bootstrap provides a way to draw probability-based, assumption-free inference for a statistic of interest.^10^ Operationally, bootstrapping involves repeatedly taking a random sample of size *n* with replacement from an original sample of size *n*, and computing a statistic of interest θ (e.g. sensitivity, specificity, and predictive values). Because the sampling is done with replacement, some observations may appear more than once and other observations may not be selected. The process of drawing a new sample and computing the statistic of interest is performed *B* times (e.g. 1000 times) to generate *B* estimates of θ. From this large number of θ estimates, the median is taken as the estimate of θ and the nonparametric CIs (e.g., 95% CI) use the 2.5th and 97.5th percentiles of the ordered distribution of the θ*s*.

For the cluster bootstrap of correlated eye data, the subjects need to be stratified by both the number of study eyes per subject (e.g. 1 or 2) and by the number of eyes with the ocular disease of interest (e.g. 0, 1, or 2). For each stratum, the first step is to randomly select the same number of subjects with replacement as the number of subjects in a given stratum.^11^ For each subject selected from sampling with replacement, all eligible eyes of the selected subjects are included in the bootstrapped sample. The desired statistic is computed using the bootstrapped sample and the process is repeated *B* times. The nonparametric CIs can be derived in the same way as the standard bootstrapping procedure. The SAS code for the cluster bootstrap for sensitivity and specificity is given in Appendix 3.

As described previously, for studies that oversampled subjects with disease, the PPV and NPV cannot be calculated directly from the study data. Instead, the PPV and NPV of an ocular test should be calculated based on its sensitivity, specificity, and the disease prevalence in the population in which the ocular test will be administered. For the cluster bootstrap of PPV and NPV, the sensitivity and specificity will be calculated first from each bootstrap sample, then PPV and NPV will be calculated based on the calculated sensitivity, specificity, and the assumed prevalence. The nonparametric CIs for PPV and NPV are derived from their empirical distributions over many (B) bootstrap samples. The SAS code for the cluster bootstrap for PPV and NPV is given in Appendix 4.

### Determination of Test Ocular Performance at the Person Level

In some situations, although the ocular test is performed in both eyes of a subject, calculating sensitivity, specificity, and predictive values at the person level (i.e. using the person as the unit of analysis) may be more relevant than calculating sensitivity and specificity at the eye level. For example, when screening for ocular disease, a subject may be referred when the test is positive for one or both eyes. For a person-level analysis, we define ocular disease present in a subject if ocular disease is present in either eye, and we define the test positive in a subject if the ocular test is positive in either eye. After the person-level data are derived, the sensitivity, specificity, predictive values, and their 95% CIs can be calculated using the standard method as described previously for independent data. When sensitivity and specificity of a test are analyzed in this way, the person level sensitivity will be higher and the specificity lower than when the test accuracy is assessed per eye.

### Example: Telemedicine System for the Evaluation of Acute-phase Retinopathy of Prematurity 

The evaluation of acute-phase retinopathy of prematurity (e-ROP) study was a multicenter study to evaluate the validity of a telemedicine system for identifying infants who have sufficiently severe retinopathy of prematurity (called referral-warranted ROP [RW-ROP]) to require evaluation by an ophthalmologist.^12^ The study enrolled 1257 premature infants and each infant underwent a regularly scheduled diagnostic examination by an ophthalmologist and digital imaging by a nonphysician imager. Ophthalmologists documented findings consistent with RW-ROP (defined as presence of either zone I ROP, ROP stage 3 or higher, or plus disease). Masked nonphysician readers graded a standard 6-image set per eye for ROP stage, zone, and presence of plus disease. The validity of the telemedicine system was evaluated using sensitivity and specificity by comparing the image evaluation (ocular test) findings to the ophthalmologist clinical examination findings (reference standard).

For the purpose of demonstration, we selected an enriched (higher prevalence of RW-ROP) sample of 100 infants that included 29 infants with RW-ROP in either eye and 71 infants without RW-ROP as determined based on clinical eye examination. The sensitivity and specificity were calculated using data from one session of digital image/clinical eye examination from each infant. For infants with RW-ROP based on the clinical eye examination, the session when the results of the clinical examination are first identified as RW-ROP were used. For infants without RW-ROP, a session was selected randomly. At the same selected session, we compared the RW-ROP finding from evaluation of an image set (positive or negative) to the RW-ROP finding of the clinical eye examination (presence or absence). We calculated sensitivity, specificity and their 95% CIs per-eye and per-infant, with per-eye analysis as the primary and per-infant analysis as the secondary end point as executed in the e-ROP study.^12^ For the per-eye analysis, the inter-eye correlation was accommodated by using both GEE and cluster bootstrap approaches. In the cluster bootstrap, because each infant contributed both eyes for the study, infants were divided into 3 strata including 1 stratum for 71 infants without RW-ROP in both eyes, a second stratum for 9 infants with RW-ROP only in 1 eye, and a third stratum for 20 infants with RW-ROP in both eyes. If some infants had only contributed one eye to the study, two additional strata would be formed (e.g. one stratum for infants without RW-ROP in the study eye and another stratum for infants with RW-ROP in the study eye). The SAS code for these analyses can be found in Appendix 2 for the GEE approach, and Appendix 3 for the cluster bootstrap approach.

Using the sensitivity and specificity values and the anticipated prevalence of RW-ROP, we calculated the PPV and NPV using the methods described above. In the e-ROP study, the overall RW-ROP rate was 19.4% at the infant level, but varied across neonatal intensive care units, ranging from 8.8% to 29.7%. Thus, we calculated the PPV and NPV and their 95% CIs under the assumption of prevalence of RW-ROP ranging from 5% to 30%. The sensitivity and specificity from both infant-level analysis and eye-level analysis were used for the PPV and NPV calculation. The cluster bootstrap was used for the calculation of 95% CIs of eye-level PPV and NPV. The SAS code for the calculations of NPV and PPV is in Appendix 1 for infant-level analysis and Appendix 4 for eye-level analysis using the cluster bootstrap approach.

## Results

Among 100 infants selected for analysis, 29 infants had RW-ROP in either eye based on clinical eye examination, including 20 infants with RW-ROP in both eyes and 9 infants with RW-ROP only in one eye (Table 2). Ninety-one (91%) of 100 infants were in agreement between 2 eyes in the status of RW-ROP from clinical eye examination, with Kappa of 0.76 (95% CI = 0.61–0.91).^13^

**Table 2.** Inter-Eye Agreement in RW-ROP Status From Clinical Eye Examination (*N* = 100 infants)

	Right Eye		
Left Eye	RW-ROP Absent	RW-ROP Present	Total
RW-ROP absent	71	3	74
RW-ROP present	6	20	26
Total	77	23	100
	Kappa (95% CI) = 0.76 (0.61–0.91)		

The agreement between RW-ROP status from image evaluation versus clinical eye examination is shown in Table 3 for an eye-level comparison and in Table 4 for an infant-level comparison. The sensitivity and specificity and corresponding 95% CIs from various approaches are reported in Table 5. In the per-eye analysis that included both eyes of an infant, the sensitivity was 83.7% and specificity 86.8% for both the naïve approach that ignored the inter-eye correlation and the GEE approach and cluster bootstrap that accounted for the inter-eye correlation. However, the 95% CIs from the naïve approach calculated using the exact method were narrower than those of the GEE approach and cluster bootstrap for both sensitivity (width of the 95% CI = 22.4% vs. 23.2% vs. 23.9%) and specificity (11.4% vs. 12.5% vs. 11.6%). As expected in the analysis using the naïve approach, the width of the 95% CI using the exact method is wider than using the normal approximation method for both sensitivity (width of 95% CI = 22.4% vs. 20.7%) and specificity (width of 95% CI = 11.4% vs. 10.8%). The 95% CIs for sensitivity and specificity calculated from left eyes and right eyes separately were wider (35.2% and 30.8%, respectively, for sensitivity, 25.4% and 17.3%, respectively, for specificity), reflecting the loss of information from analyzing only data from one eye. Although we have no reason to expect differences in grading performance for right and left eyes, the sensitivity from the right eye analysis was somewhat higher. Because infants contributed data from two eyes, the estimated sensitivity and specificity from the per eye analysis of all data are simply the weighted averages of the values from the separate analysis of left or right eyes, respectively. Although both the GEE and bootstrap approaches accounted for the inter-eye correlation, they provided somewhat different estimated 95% CIs for sensitivity and specificity. These differences are due to the fact that they used different methods to account for inter-eye correlation. GEE is a model-based approach, and its 95% CIs were calculated based on a working independence covariance matrix. The bootstrap we used is a nonparametric method that is based on resampling of the data to get the empirical distribution of sensitivity and specificity for deriving percentile-based 95% CIs.

**Table 3.** Cross-Tabulation of RW-ROP Status From Diagnostic Eye Examination and From Image Evaluation at the Eye-Level (*N* = 200 Eyes From 100 Infants)

	Clinical Eye Examination		
Image Evaluation	RW-ROP Absent	RW-ROP Present	Total
RW-ROP negative	131 (86.8%)	8 (16.3%)	139
RW-ROP positive	20 (13.2%)	41 (83.7%)	61
Total	151	49	200

**Table 4.** Cross-Tabulation of RW-ROP From Diagnostic Eye Examination versus Image Evaluation at Eye Level (*N* = 100 Infants)

	Clinical Eye Examination: RW-ROP Status in Left Eye/Right Eye				
Image Evaluation: RW-ROP Status in Left Eye/Right Eye	Absent/Absent	Absent/Present	Present/Absent	Present/Present	Total
Negative/negative	61	0	0	1	62
Negative/positive	4	1	**3**	1	9
Positive/negative	3	0	1	2	6
Positive/positive	3	2	2	16	23
Total	71	3	6	20	100

**Table 5.** Sensitivity and Specificity of Image Evaluation for RW-ROP Using Various Analysis Approaches

Analysis Approach	Sensitivity				Specificity			
Per-Eye Analysis	*N*	Estimate	95% CI	Width of 95% CI	*N*	Estimate	95% CI	Width of 95% CI
Naïve approach: normal approximation	49	83.7%	73.3–94.0%	20.7%	151	86.8%	81.4–92.2%	10.8%
Naïve approach: exact method	49	83.7%	70.3–92.7%	22.4%	151	86.8%	80.3–91.7%	11.4%
GEE	49	83.7%	69.0–92.2%	23.2%	151	86.8%	79.3–91.8%	12.5%
Cluster bootstrap	49	84.4%	70.8–94.7%	23.9%	151	87.0%	80.6–92.6%	11.6%
Left eye only^*^	26	80.8%	60.7–95.9%	35.2%	74	89.2%	79.8–95.2%	25.4%
Right eye only^*^	23	87.0%	66.4–97.2%	30.8%	77	84.4%	74.4–91.7%	17.3%
Per-infant analysis^*^	29	96.6%	82.2–99.9%	17.7%	71	85.9%	77.8–94.0%	16.2%

* Confidence interval was calculated using Clopper-Pearson exact method.

In the per-infant analysis that considered image evaluation as positive if RW-ROP was positive in either eye, the sensitivity was higher (96.6%), but specificity was lower (85.9%) than those from the per-eye analysis. As shown in Table 4, there are three infants who were RW-ROP positive on image evaluation only in the right eye and RW-ROP disease was present from clinical eye examination only in the left eye. For these three infants, the correct action (referral) would be made but the actual classification of the eyes would be incorrect.

The positive and negative predictive values corresponding to the sensitivity (96.6%) and specificity (85.9%) estimates from the per-infant analysis with the RW-ROP prevalence ranging from 5% to 30% are reported in Table 6. When the prevalence was 5%, the PPV was low (26.5%, 95% CI = 16.8–39.1%) and NPV was high (99.8%, 95% CI = 98.6–99.9%). However, when the prevalence was 30%, the PPV increased substantially (74.6%, 95% CI = 62.2–84.0%), whereas NPV decreased slightly (98.3%, 95% CI = 89.4–99.8%). When the same prevalence of RW-ROP ranging from 5% to 30% and the eye-level sensitivity (83.7%) and specificity (86.8%) were used to calculated eye-level PPV and NPV, PPV values were similar, whereas the NPV values were less than those from infant-level analysis (see Table 6).

**Table 6.** The Positive and Negative Predictive Values From Image Evaluation of RW-ROP at Various Prevalence

	Person-Level Analysis^*^		Eye-Level Analysis^†^	
Assumed Prevalence of RW-ROP	Positive Predictive Value (95% CI)^*^	Negative Predictive Value (95% CI)^*^	Positive Predictive Value (95% CI)	Negative Predictive Value (95% CI)
5%	26.5% (16.8–39.1%)	99.8% (98.6–99.9%)	25.4% (18.3–36.0%)	99.1% (98.3–99.6%)
10%	43.2% (29.9–57.6%)	98.6% (97.0–99.9%)	41.3% (31.9–54.5%)	98.0% (96.5–99.3%)
15%	54.7% (40.4–68.3%)	99.3% (95.3–99.9%)	52.8% (42.5–65.8%)	96.9% (94.6–98.8%)
20%	63.1% (49.0–75.3%)	99.0% (93.5–99.9%)	61.3% (51.7–72.7%)	95.7% (92.4–98.3%)
25%	69.5% (56.1–80.3%)	98.7% (91.6–99.8%)	68.3% (57.8–78.2%)	94.3% (90.0–97.8%)
30%	74.6% (62.2–84.0%)	98.3% (89.4–99.8%)	73.1% (64.7–82.4%)	92.7% (87.9–97.1%)

* Predictive values were calculated by using sensitivity of 96.6% and specificity of 85.9% from the per-infant analysis in Table 4, their 95% CIs were calculated using the logit transformation.

† Predictive values were calculated by using sensitivity of 83.7% and specificity of 86.8% from the eye-level analysis in Table 5, and their 95% CIs were calculated using the cluster bootstrap.

## Conclusion

The performance of an ocular test is evaluated using sensitivity, specificity, and predictive values. When an ocular test is performed in both eyes of some or all of the study subjects, the statistical analyses for the validity of an ocular test are best performed at the eye-level and account for the inter-eye correlation by using either the GEE or cluster bootstrap to provide accurate coverage of the confidence interval. Ignoring the inter-eye correlation results in 95% CIs that are inappropriately narrow. Although analyzing data from two eyes separately avoids the need to account for the inter-eye correlation, such analyses are not efficient, producing CIs that are wider than when both eyes are analyzed. When ocular testing results will ultimately lead to a person-level decision (e.g. when the ocular test is positive in either eye, the person will be referred for diagnostic examination in both eyes), the evaluation of an ocular test can be supplemented by person-level analyses for sensitivity, specificity, and predictive values.

## Supplementary Material

Supplement 1
