# Supplementary material for: Calculating Sensitivity, Specificity, and Predictive Values for Correlated Eye Data
Source: Invest Ophthalmol Vis Sci. 2020 Sep 16;61(11):29. doi: 10.1167/iovs.61.11.29 (PMC7500131; doi:10.1167/iovs.61.11.29)
Supplement: Supplement 1 [file iovs-61-11-29_s001.pdf]

Appendix 1: SAS macro for calculating the PPV, NPV and their 95% confidence Intervals at the person-level

```
/****************************************************************************
Calculate the PPV, NPV and their 95% CI using the Wald-type formulation
based on the paper by Mercaldo ND et al, Confidence Intervals for Predictive
values with an emphasis to case-control studies. Statistics in Medicine
2007;26:2170-2183. The parameters needed for the Macro are:
Se=Sensitivity
Sp=Specificity
n1=# of subjects with disease of interest that the sensitivity calculation
was based on
n0=# of controls without disease of interest that the specificity calculation
was based on
p=Prevalence of disease in the population that the diagnostic test will be
applied to
****************************************************************************/
%macro PPV_NPV(Se=, Sp=, n1=, n0=, p=);

data PPV_NPV;
 Se=&se;
 Sp=&sp;
 n1=&n1;
 n0=&n0;
 p=&p;
 PPV=(Se*p)/(Se*p+(1-Sp)*(1-p));
 NPV=(Sp*(1-p))/((1-Se)*p+(Sp*(1-p)));


  /*** for 95% CI of PPV **/
 var_PPV_nu=(p*(1-Sp)*(1-p))**2*Se*(1-Se)/n1 +(p*Se*(1-p))**2*Sp*(1-Sp)/n0;
 var_PPV_de=(Se*p+(1-Sp)*(1-p))**4;
 var_PPV= var_PPV_nu/var_PPV_de;

 PPV_lower=max(0, PPV-1.96*sqrt(var_PPV));
 PPV_upper=min(1, PPV+1.96*sqrt(var_PPV));


  /*** for 95% CI of NPV **/
 var_NPV_nu=(Sp*(1-p)*p)**2*Se*(1-Se)/n1 + ((1-Se)*(1-p)*p)**2*Sp*(1-Sp)/n0;
 var_NPV_de=((1-Se)*p+Sp*(1-p))**4;
 var_NPV= var_NPV_nu/var_NPV_de;

 NPV_lower=max(0, NPV-1.96*sqrt(var_NPV));
 NPV_upper=min(1, NPV+1.96*sqrt(var_NPV));


  /***** calculate 95% CI using logit transformation *****/
logit_PPV=log(PPV/(1-PPV));
logit_NPV=log(NPV/(1-NPV));
var_logit_PPV=((1-Se)/Se)*(1/n1)+(Sp/(1-Sp))*(1/n0);
var_logit_NPV=(Se/(1-Se))*(1/n1)+((1-Sp)/Sp)*(1/n0);
logit_PPV_lower=logit_PPV-1.96*sqrt(var_logit_PPV);
logit_PPV_upper=logit_PPV+1.96*sqrt(var_logit_PPV);
```

```
logit_NPV_lower=logit_NPV-1.96*sqrt(var_logit_NPV);
logit_NPV_upper=logit_NPV+1.96*sqrt(var_logit_NPV);

PPV_logit_lower=exp(logit_PPV_lower)/(1+exp(logit_PPV_lower));
PPV_logit_upper=exp(logit_PPV_upper)/(1+exp(logit_PPV_upper));

NPV_logit_lower=exp(logit_NPV_lower)/(1+exp(logit_NPV_lower));
NPV_logit_upper=exp(logit_NPV_upper)/(1+exp(logit_NPV_upper));

run;

proc print data=PPV_NPV;
 var PPV PPV_Lower PPV_upper NPV NPV_lower NPV_upper
      PPV_logit_Lower PPV_logit_upper NPV_logit_lower NPV_logit_upper;
run;

%mend;
```

Appendix 2: SAS macro for calculating the sensitivity, specificity and their 95% CI at the eye level using GEE approach

```
/***************************************************************************
Calculate the sensitivity, specificity and their 95% CI using the GEE
approach for the per-eye analysis
The input variables need for the macro:
Data: The eye-specific SAS data file that has subject ID (ID), disease status
(disease), and the diagnostic test result (test).
Disease: The status of disease from the reference stand examination.
         1=Presence of disease, 0=Absence of disease
Test: The diagnostic test result
      1=Test positive, 0=Test negative
***************************************************************************/

%macro gee(data=, disease=, test=);

proc genmod data=&data descending;
 class id &disease;
 model &test=&disease/dist=bin;
 repeated subject=id/type=ind;
 estimate 'Sensitivity' intercept 1 &disease 0 1/exp;
 estimate 'Spec.ficity' intercept 1 &disease 1 0/exp;
 ods output Genmod.Estimates=sensdata;
run;

data CI;
 set sensdata (rename=(LBetaestimate=estimate LBetaLowerCL=LowerCL
LBetaUpperCL=UpperCL));
 if label='Exp(Sensitivity)' then do;
     point=estimate/(1+estimate);
     lower=lowerCL/(1+lowerCL);
     upper=upperCL/(1+upperCL);
  end;

 if label='Exp(Specificity)' then do;
     point=1/(1+estimate);
     upper=1/(1+lowerCL);
     lower=1/(1+upperCL);
 end;
 if label in ('Exp(Sensitivity)', 'Exp(Specificity)');
run;

proc print data=ci;
 var label point lower upper;
run;
%mend;

%gee(data=subsample, disease=RWROP_de, test=RWROP_rc);
```

Appendix 3: SAS Macro for calculating the sensitivity, specificity and their 95% CI at the eye level using cluster bootstrap approach

```
/****************************************************************************
Calculate the sensitivity, specificity and their 95% CI using the cluster
bootstrap approach for the per-eye analysis of e-ROP data
The input variables need for the macro:
Data: The eye-specific SAS data file that has subject ID (ID), eye, disease
status (disease), and the diagnostic test result (test).
Disease: The status of disease from the reference standard examination.
         1=Presence of disease, 0=Absence of disease
Test: The diagnostic test result
      1=Test positive, 0=Test negative
B: The number of cluster bootstrap samples
****************************************************************************/

%macro boot_sens(pdata=, pind=, edata=, b=1000);
data orig0 (where=(count=0) keep=id count)
     orig1 (where=(count=1) keep=id count)
     orig2 (where=(count=2) keep=id count);
   set &person;
run;

data bootpat;
   call streaminit(02262020);
   %do t=0 %to 2;
   %let dsnid=%sysfunc(open(orig&t));
   %let nobs=%sysfunc(attrn(&dsnid,nlobs));
    do sample=1 to &b;
      do i = 1 to &nobs;
            pt = round(ranuni(&t)*&nobs);
         set orig&t point=pt;
         output;
        end;
    end;
   %end;
stop;
run;

data bootpat_eye;
 set bootpat;
 eye='0';
 output;
 eye='1';
 output;
run;

proc sort data=bootpat_eye; by id eye;
proc sort data=&edata; by id eye;

data boot;
 merge &edata(in=aa) bootpat_eye(in=bb); by id eye;
 if bb;
run;

proc sort data=boot; by sample;
```

```
ods listing close;
proc freq data=boot;
 tables RWROP_DE*RWROP_RC/nocol nopercent;
 format RWROP_RC yesnof.;
 ods output CrossTabFreqs=freq;
 by sample;
run;

data sens_spec(keep=sample sens spec);
 set freq; by sample;
 retain sens spec;
 if first.sample then do; sens=.; spec=.; end;
 if RWROP_DE=1 and RWROP_RC=1 then sens=rowpercent;
 if RWROP_DE=0 and RWROP_RC=0 then spec=rowpercent;
 if last.sample;
run;

proc sort data=sens_spec; by sens;
data sens_CI;
  set sens_spec end=eof nobs=nobs;
  retain sens95_low sens_med sens95_hi;
  if _n_= nobs*0.975 then sens95_hi=sens;
  if _n_= nobs*0.5 then sens_med=sens;
  if _n_= nobs*0.025 then sens95_low=sens;
  if eof then output;
run;

proc means data=sens_spec mean std median;
 var sens spec;
run;

proc print data=sens_CI;
 var sens95_low sens_med sens95_hi;
  title "95% CI for Sensitivity";
run;

proc sort data=sens_spec; by spec;

data spec_CI;
  set sens_spec end=eof nobs=nobs;
  retain spec95_low spec_med spec95_hi;
  if _n_= nobs*0.975  then spec95_hi=spec;
  if _n_= nobs*0.5 then spec_med=spec;
  if _n_= nobs * 0.025 then spec95_low=spec;
  if eof then output;
run;

ods listing;
proc print data=spec_CI;
 var spec95_low spec_med spec95_hi;
  title "95% CI for Specificity";
run;
%mend;

%boot_sens(pdata=person, pind=count, edata=eROP, b=2000);
```

Appendix 4: SAS macro for 95% CI for positive predictive value and negative predictive value using cluster bootstrap approach

```
/************************************************************************
Calculate the 95% CI of PPV and NPV using the cluster bootstrap approach for 
the per-eye analysis of e-ROP data
The input variables need for the macro:
Pdata: The infant-level data that has the count of how many eyes had RW-ROP 
in each infant.
Edata: The eye-specific SAS data file that has subject ID (ID), eye, disease 
status (disease), and the diagnostic test result (test).
Pind: the
P: the prevalence of RW-ROP in the anticipated population
B: The number of cluster bootstrap samples
************************************************************************/

%macro boot_PV(pdata=, edata=, p=, b=1000);
data orig0 (where=(count=0) keep=id count)
     orig1 (where=(count=1) keep=id count)
     orig2 (where=(count=2) keep=id count); /* Create one data set for each 
RW-ROP count group (0, 1, 2) */
   set &pdata;
run;

data bootpat;
   call streaminit(02262020);
   %do t=0 %to 2;
   %let dsnid=%sysfunc(open(orig&t));
   %let nobs=%sysfunc(attrn(&dsnid,nlobs));
    do sample=1 to &b;
      do i = 1 to &nobs;
            pt = round(ranuni(&t)*&nobs);
         set orig&t point=pt;
         output;
        end;
    end;
   %end;
stop;
run;

data bootpat_eye;
 set bootpat;
 eye='0';
 output;
 eye='1';
 output;
run;

proc sort data=bootpat_eye; by id eye;
proc sort data=&edata; by id eye;

data boot;
 merge &edata(in=aa) bootpat_eye(in=bb); by id eye;
 if bb;
run;

proc sort data=boot; by sample;
```

```
ods listing close;
proc freq data=boot;
 tables RWROP_DE*RWROP_RC/nocol nopercent;
 format RWROP_RC yesnof.;
 ods output CrossTabFreqs=freq;
 by sample;
run;

data sens_spec(keep=sample sens spec);
 set freq; by sample;
 retain sens spec;
 if first.sample then do; sens=.; spec=.; end;
 if RWROP_DE=1 and RWROP_RC=1 then sens=rowpercent;
 if RWROP_DE=0 and RWROP_RC=0 then spec=rowpercent;
 if last.sample;
run;

proc means data=sens_spec mean std median;
 var sens spec;
run;

data PV;
 set sens_spec;
 PPV=(Sens/100*&p)/(Sens/100*&p+(1-Spec/100)*(1-&p));
 NPV=(Spec/100*(1-&p))/((1-Sens/100)*&p+(Spec/100*(1-&p)));
run;

proc sort data=PV; by PPV;
data PPV_CI;
  set PV end=eof nobs=nobs;
  retain PPV95_low PPV_med PPV95_hi;
  if _n_= nobs*0.975  then PPV95_hi=PPV;
  if _n_= nobs*0.5 then PPV_med=PPV;
  if _n_= nobs * 0.025 then PPV95_low=PPV;
  if eof then output;
run;

proc means data=PV mean std median;
 var PPV NPV;
run;

proc print data=PPV_CI;
 var PPV95_low PPV_med PPV95_hi;
  title "95% CI for Postive Predictive Value";
run;

proc sort data=PV; by NPV;

data NPV_CI;
  set PV end=eof nobs=nobs;
  retain NPV95_low NPV_med NPV95_hi;
  if _n_= nobs*0.975 then NPV95_hi=NPV;
  if _n_= nobs*0.5 then NPV_med=NPV;
  if _n_= nobs * 0.025 then NPV95_low=NPV;
  if eof then output;
run;
```

```
ods listing;
proc print data=NPV_CI;
 var NPV95_low NPV_med NPV95_hi;
  title "95% CI for Negative Predictive Value";
run;
%mend;

%boot_PV(pdata=sub_person, edata=subsample, P=0.05, b=2000);
```
